# Development and validation of the patient roles and responsibilities scale in cancer patients

**DOI:** 10.1007/s11136-018-1940-2

**Published:** 2018-07-26

**Authors:** Valerie Shilling, Rachel Starkings, Valerie Jenkins, David Cella, Lesley Fallowfield

**Affiliations:** 1 Sussex Health Outcomes Research and Education in Cancer (SHORE-C), Brighton and Sussex Medical School, University of Sussex, Falmer, Brighton, UK; 2 Department of Medical Social Sciences, Northwestern University Feinberg School of Medicine, Chicago, IL USA

**Keywords:** Cancer, Outcome measures, Psychometric performance, Validation, Questionnaire development

## Abstract

**Purpose:**

The Patient Roles and Responsibilities Scale (PRRS) was developed to enable a broader evaluation of the impact of cancer and cancer treatment, measuring ‘real world’ roles and responsibilities such as caring for others and financial and employment responsibilities. Here, we report the development and initial validation.

**Methods:**

The 29-item PRRS was developed from the thematic analysis of two interview studies with cancer patients and caregivers. In the evaluation study, participants completed the PRRS alongside the Social Difficulties Inventory (SDI), the main criterion measure for concurrent validity, and the Functional Assessment of Cancer Therapy - General and WHO Quality of Life-BREF (WHOQOL-BREF) for additional convergent validity data. Questionnaires were completed at baseline, 7-days (PRRS only) and 2 months. Demographic data and patient characteristics were collected at baseline.

**Results:**

One hundred and thirty-five patients with stage III/IV breast, lung or gynaecological cancer or melanoma completed the PRRS at least once. Five items performed poorly and were removed from the scale. The final 16 core items selected comprised 3 dimensions: family well-being, responsibilities and social life, and financial well-being, identified in principal component analysis, accounting for 61.5% of total variance. Missing data (0.6%) and floor/ceiling effects were low (0%/1.5%). Cronbach’s alpha was 0.9 for the PRRS-16; 0.79–0.87 for the subscales. PRRS showed good test–retest reliability (ICC-0.86), sensitivity to change and the predicted pattern of correlation with validation measures *r* = |0.65–0.77|. The standalone 7-item jobs and careers subscale requires further validation.

**Conclusions:**

Initial evaluation shows that the PRRS is psychometrically robust with potential to inform the evaluation of new treatments in clinical trials and real-world studies.

**Electronic supplementary material:**

The online version of this article (10.1007/s11136-018-1940-2) contains supplementary material, which is available to authorized users.

## Background

More patients with cancer are living longer due in part to advances in molecular biology and the development of novel-targeted therapies [1]. There is growing recognition that assessment of the quality of this extended survival is important, namely how broader aspects of patients’ lives are managed and affected. Quality of survival (QoS) looks beyond traditional endpoints of survival times, response and adverse events towards an understanding of the longer term patient experience. Topics include emotional concerns, financial burdens and impacts on role functioning [2]. Despite the extended survival benefits, associated side-effects from novel treatments can impact on a patient’s ability to live a ‘normal’ life. With maintenance therapy, patients may face a trade-off between the putative extension and quality of that survival [3]. Uncertainties about likely survival benefit can leave patients and their families unable, or unwilling to make plans for the future [4].

Well-validated measures of financial and social distress exist (Comprehensive Score for Financial Toxicity [5, 6] and Social Difficulties Inventory [7, 8]), but there are currently no such measures that capture fully all aspects of the concept of QoS. The Quality of Life in Adult Cancer Survivors (QLACS) measure covers a number of these areas e.g. financial problems, family-related distress, but was developed specifically for use with long-term cancer survivors, defined by the authors as greater than five years from diagnosis [9]. Although the European Organisation for Research and Treatment of Cancer (EORTC) and the National Institute of Health (NIH) funded Patient Reported Outcomes Measurement Information System (PROMIS) initiatives have developed role functioning item banks for use in computer adaptive testing [10, 11], the impact of disease and treatment on roles and responsibilities is generally underrepresented in health-related patient reported outcome measures (PROMs). This is a serious oversight as many patients have caregiving responsibilities, for children, grandchildren, parents, partners or even pets. Qualitative studies demonstrate that these types of roles and responsibilities outside of work are a significant part of patients’ lives, which cancer and its treatment can seriously impinge upon [12–16]. As role functioning issues are somewhat poorly described, many patients feel that information on how to cope at home is inadequate [17].

Our recent systematic reviews of PROMs currently in use identified areas of concern that are insufficiently researched, namely family, financial and occupational circumstances [18, 19]. Furthermore, QoS is not a static concept; it includes recognition that the relative importance of different dimensions will change across the disease trajectory. As such we designed the PROACT programme of work (Patient Reported Outcomes impact of Age and Carer role demands associated with Treatment) recognising that patients and their families are continually adjusting to a fluid situation whilst trying to maintain their ‘real world’ roles and responsibilities beyond cancer, such as caregiving responsibilities for a spouse or children/grandchildren, jobs and financial responsibilities [4].

## Overview

PROACT is a multi-phase project, informed by the QoS conceptual framework, with the primary aim to develop and evaluate two self-report measures; one for patients and another for informal caregivers to assess comprehensively broader impacts of disease and treatment. The measures have been developed to be compatible with the Functional Assessment of Chronic Illness Therapy (FACIT) measurement system and are intended to benefit future patients and caregivers by capturing these wider impacts of cancer and its treatment during the evaluation of new treatments in clinical trials and could be used to aid clinical conversations around treatment and intervention.

To develop the PROACT measures, we conducted two systematic reviews [18, 19] to inform our understanding of the content and psychometric properties of measures currently being used, and to identify gaps in relevant domains. Development of the two PROACT measures then followed three phases of scale development: item generation (study 1), item-reduction and scale construction (study 2) and initial scale evaluation and validation (study 3). Because the topics covered in the scale are not related to specific issues such as treatment side effects, rather to the broader impact such as time off work, it is intended that it will be useful for all groups of cancer patients. The development studies were conducted with patients with stage III/IV cancer and the person they nominated as their main source of support. However, the measures are intended to be suitable for all stages of cancer and are currently being validated in early stage disease. This paper reports the development and evaluation of the multi-dimensional Patient Roles and Responsibilities Scale (PRRS). The Caregiver Roles and Responsibilities Scale (CRRS) was developed concurrently and is reported elsewhere.

## Ethics statement

Studies received ethics approval from London Queen Square Research Ethics Committee (ref: 15/LO/1323 Studies 1 and 2; ref: 16/LO/1125 Study 3). Signed informed consent was obtained from all participants.

## Eligibility criteria

Inclusion/exclusion criteria were similar across all three studies. Eligible patients had advanced (stage III/IV) melanoma, lung or gynaecological cancer, with a requirement that they could nominate an informal caregiver also willing to take part. Both patients and caregivers were required to be over 18 years of age, have capacity to give informed consent and be able to read and speak English. Those who were currently inpatients or acutely distressed for any reason were excluded. In studies 2 and 3, we expanded the patient population to include women with breast cancer. The cancer types were selected to represent a range of potential cancer experiences in order that the measure might be sufficiently generic for other tumour sites. Study 3 did not require that both patient and caregiver consent to the study; either party could participate alone.

## Scale development: methods and results

### Study 1: item generation

We conducted in-depth qualitative interviews with 24 patients with advanced cancer and separate interviews with their nominated informal caregivers (*N* = 23) about the impact of extended cancer survival on broader aspects of life and well-being (see Supplementary File S1 for participant demographics). Topic guides were informed by our earlier systematic reviews [18, 19] and through discussion with advisors with a lived experience of cancer or who were supporting someone with cancer. Participants were first invited to generate and speak freely about topics important to their well-being and if and how these areas of life had been impacted by their/their loved one’s cancer diagnosis and treatment. Further discussion was prompted (where necessary) around topics such as family life, relationships, leisure and social activities, employment and finances, physical and emotional health. Interviews were recorded (38.5 h of recorded data) and transcribed verbatim.

A thematic framework was developed from an initial process of open coding and tested iteratively as new data were collected. Thematic analysis identified 20 themes and 33 sub-themes from which a long list of 179 potential items was generated for the patient scale. These were reviewed for relevance and redundancy by the authors before 44 potential items were reviewed by our panel of advisors with lived experience. Consensus decisions on whether an item was retained or not were made through discussion informed by the thematic analysis of the interviews. Items were evaluated based on the relative importance the content appeared to have across interviews both in terms of the frequency with which the topic was discussed by participants and the significance attached to it.

Thirty items were retained for evaluation in Study 2. Where appropriate items already existed in the FACIT system, these validated items were used, rather than generating new ones. This included three items from the FACIT Comprehensive Score for Finacial Toxicity (COST) measure [5].

Interviews and thematic analysis were conducted by VS/RS. The long list of potential items was generated by VS/RS/VJ/LF and reviewed and reduced through discussion between VS/RS/VJ/LF and our panel of five advisors with lived experience.

### Study 2: item reduction and scale construction

Cognitive interview techniques were employed to ‘test’ the potential questionnaire items. The processes participants applied when answering were explored, enabling us to check that the items were being understood as intended and consistently by different people, for example [20]. Cognitive interviews were conducted with a new cohort of 20 patients and informal caregivers using a mixture of the ‘think aloud’ technique (where participants verbalise the thought processes they go through in answering a questionnaire item) and specific probes around comprehension, retrieval, judgement and response options to assess each of the potential scale items. Example probes included: *What does that question mean to you, in your own words*? *What factors did you consider when you were deciding on your answer*? *Were you able to match the answer you wanted to give to one of the choices we’ve given you*? Items were also discussed in terms of acceptability, relevance, redundancy and importance. Participants were also asked to identify missing topics. See Supplementary File S1 for participant demographics.

Scale items were revised, added and removed in an iterative fashion through the course of the study. Through this process 51 changes were made to the scale including 44 wording revisions, 4 deletions and 3 additions, resulting in a 29-item scale grouped conceptually into items around family and home life (*N* = 13), financial well-being (*N* = 8) and jobs and careers (*N* = 8).

As with study 1, interviews and initial analysis were conducted by VS/RS with any changes to the scale in the course of the study agreed by consensus between VS/RS/VJ/LJ after discussion of the interview content. Final scales were reviewed and agreed by VS/RS/VJ/LF and our panel of five advisors with lived experience.

### Initial evaluation and validation: study 3

#### Methods

##### Population and procedure

Participants were recruited from 11 sites in the United Kingdom, stratified by age group (≤ 50, 51–65, ≥ 66 years) and tumour site (breast, gynaecological, lung, melanoma). All participants had stage III/IV cancer. Participants completed questionnaires at home either on paper or online, whichever their preference. Demographics and the full validation pack were completed at baseline, the PRRS was completed alone after 7 days (for test–retest reliability) and the full battery completed again after 2 months (for sensitivity to change).

##### Measures

Participants completed the PRRS alongside the FACT-G, the SDI and the WHOQOL-BREF and provided basic demographic information such as age, employment status, level of education, relationship status and caregiving responsibilities along with information regarding diagnosis and treatment.

*The PRRS* The PRRS as completed in the validation study comprised 29 items: 21 core items formatted for the FACIT measurement system (responses as item applies to the past 7 days, 5 response options ranging from not at all—very much) and 1 binary response item on whether the participant has stopped work due to illness. A standalone scale, jobs and careers comprising 7 items is completed only by participants currently in paid employment (including those on sick leave). Negatively worded items were reverse scored so that a higher score corresponds to better quality of life. Where missing data occurred, total scores were prorated as long as more than half of the scale items were completed.

*The SDI* [7] The SDI is a 21-item questionnaire developed for use in oncology practice. It covers a range of difficulties that might be experienced by patients in their everyday life and includes an everyday living subscale; money matters subscale and self and others subscale. There are five standalone items about difficulties around: sex, plans to have a family, living conditions, holidays and ‘other’. Items are rated on a 4-point scale of no difficulty—very much (scored 0–3). For this analysis, we use the SD-16 scale which excludes the standalone items. The SD-16 generates a total score with range 0–44 (4 items are scored 0–2) with higher scores indicating greater social difficulty and also provides an established cut-off point to categorise patients as distressed/not distressed with the threshold set at SD-16 ≥ 10 [21].

*The FACT-G* [22] The FACT-G is a well-validated measure of health-related quality of life with 27 items comprised of four subscales: physical well-being (7 items), social well-being (7 items), emotional well-being (6 items) and functional well-being (7 items). Items are rated on a 5-point scale of not at all—very much (scored 0–4). Total score range is 0–108 with higher scores indicating higher quality of life.

Item GF7 “I am content with the quality of life right now” is used as the anchor variable to assess PRRS sensitivity to change.

*The WHOQOL-BREF* [23] The WHOQOL-BREF has 26-items producing a quality of life profile with four domain scores (physical health, psychological health, social relationships and environment) and two individually scored items about an individual’s overall perception of quality of life and health. Each item is rated on a 5-point scale (scored 1–5). The four domain scores are scaled in a positive direction with higher scores indicating a higher quality of life.

## Data analysis

Analyses were conducted using the Statistical Package for Social Sciences (IBM SPSS; version 23). Missing data from PRRS were managed by pro-rating total and were appropriate subscale scores. Missing data from other questionnaires were managed as per the specific instrument guidance. We used guidelines from the International Society for Pharmacoeconomics and Outcomes Research (ISPOR) [24, 25] and COSMIN (Consensus-based Standards for the selection of health Measurement Instruments) [26] in the development and evaluation of this measure.

### Content validity

The PRRS was developed through extensive qualitative interviews in studies 1 and 2 and through collaboration with advisors with lived experience of cancer or caring for someone with cancer; no further content validation was undertaken.

### Acceptability and precision

Acceptability was assessed by consideration of the amount of missing data overall and per item and the time to complete the scale. 15% missing data were set as the acceptable threshold for individual items.

Precision was assessed on a number of dimensions. Individual items with floor/ceiling effects > 70% were considered for removal as were those with a z-score of skewness >ǀ4ǀ. Individual items were also evaluated in terms of the pattern of correlation (items failing to show a pattern of correlation above 0.3 with multiple items or correlation with another item at ≥ 0.8 were reviewed) and of corrected item-total correlation (items with CITC < 0.3 were reviewed). Items that performed poorly on any of these criteria were considered for removal from the scale.

### Exploratory factor analysis

Principal components analysis with oblique rotation was performed to identify underlying factors. Bartlett’s test of sphericity and the Kaiser–Meyer–Olkin (KMO) value were checked to confirm that the data were suitable for factor analysis. We applied the criterion of Eigenvalue > 1.0 and also examined the scree plot to determine the number of factors retained. Items were included in the factor on which they loaded highest (minimum accepted 0.4). The unidimensionality of subscales was assessed with corrected item-total correlations (*r* ≥ 0.3 considered acceptable) and reliability with Cronbach’s alpha. Missing values were managed using listwise deletion. Finally, we assessed whether the solution made conceptual and practical sense.

### Internal consistency

Internal consistency was assessed using Cronbach’s alpha (for good internal consistency we would expect α ≥ 0.70).

### Criterion and convergent validity

Criterion validity was assessed by correlation with a commonly used legacy measure of the same concept, the SDI. We predicted a strong, negative correlation (*r* > − 0.7). Convergent validity was further evaluated by correlating with other similar measures. We predicted a moderate to strong positive correlation (0.5 < *r* < 0.7) between PRRS scores and the FACT-G and WHOQOL-BREF.

### Test–retest reliability

Test–retest reliability was assessed by comparing responses at baseline and at 7 days. Intraclass Correlations (ICCs; two-way random, absolute agreement) were calculated for total PRRS scores and sub-scale totals. An ICC > 0.7 is considered adequate, > 0.9, excellent. Weighted kappa scores (using linear weighting) were calculated for individual items; a score of *κ* ≥ 0.4 was considered acceptable, *κ* ≥ 0.6, good.

### Sensitivity to change

PRRS change scores were calculated (T1–T3). Patients were categorised as improved, worsened or unchanged based on responses to the anchor question ‘I am content with the quality of my life right now’. Paired *t* tests were used to determine if PRRS change within a group was significantly different from zero. Spearman correlation coefficients were calculated to explore the relationship between changes on the PRRS, anchor variable and other validation measures. We would expect moderate positive correlation (0.3 < *r* < 0.5) between change on the PRRS and the FACT-G and WHOQOL-BREF and moderate negative correlation between change on the PRRS and SDI (− 0.3 < *r* < − 0.5).

## Results

### Participants

**Fig. 1:**
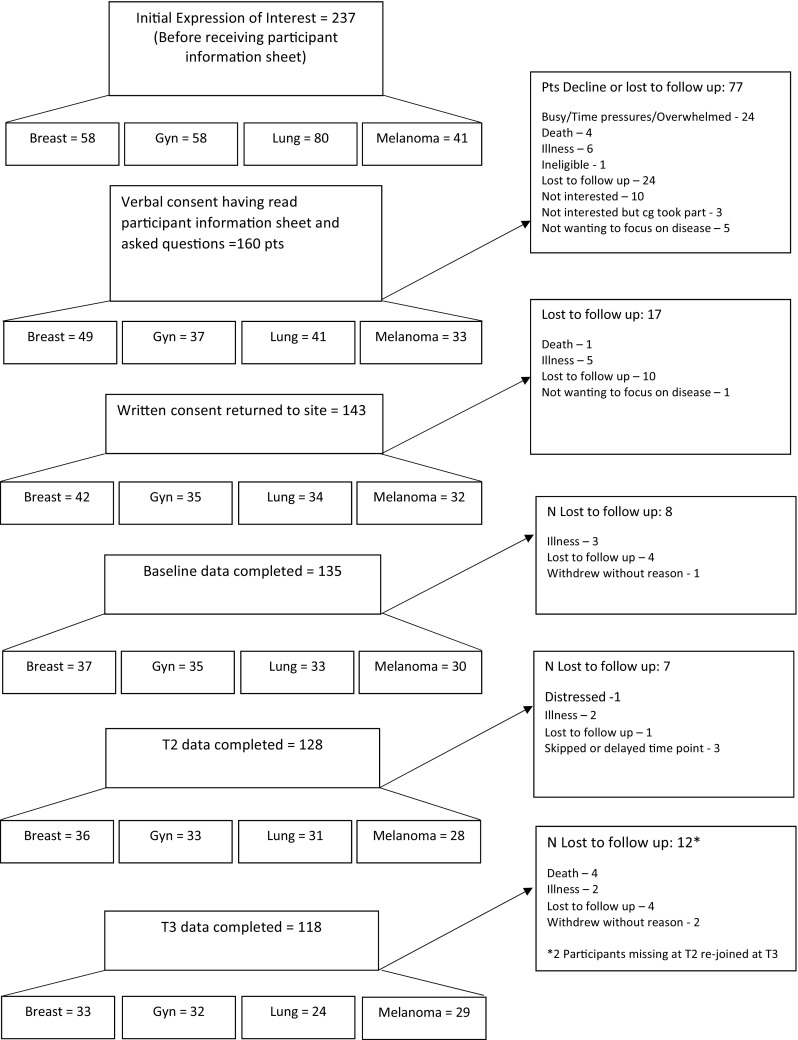
Flow of participants through study

One hundred and forty-three patients consented to take part, 135 completed baseline (37 breast, 35 gynaecological, 33 lung, 30 melanoma), 128 completed T2 and 118 completed T3. At baseline age ranged from 33 to 85 (median 61 years), 77% were female. See Fig. 1 for full details of patients approached, consented and completed questionnaires at each time point, along with reasons for decline/drop out and Table 1 for key participant characteristics.

**Table 1:** Key patient characteristics^a^ and PRRS total scores^b^

	*N* = 135	PRRS (Mean ± SD)	Univariate *P*
Cancer type			
Breast	37 (27.5%)	36.00 ± 14.38	0.060
Gynaecological	35 (26%)	40.07 ± 12.54	
Lung	33 (24.5%)	37.36 ± 11.28	
Melanoma	30 (22%)	44.13 ± 12.71	
Time since diagnosis			
<1 year	40 (30%)	36.61 ± 12.44	0.329
1–2 years	21 (15%)	40.48 ± 15.22	
>2 years	74 (55%)	40.23 ± 12.69	
On treatment (missing *N* = 22)			
Yes	76 (67%)	40.16 ± 12.74	0.445
No	37 (33%)	38.14 ± 13.99	
Age group			0.003 Post hoc comparison (Bonferroni) old vs young *p* = .003
≤ 50	34 (25%)	34.54 ± 11.73	
51–65	55 (41%)	38.03 ± 12.68	
≥ 66	46 (34%)	44.03 ± 13.07	
Sex			
Female	104 (77%)	38.25 ± 13.52	0.121
Male	31 (23%)	42.39 ± 10.90	
Relationship status			
Partner	105 (78%)	39.39 ± 13.03	0.754
No partner	30 (22%)	38.53 ± 13.28	
Employment status			
Retired	70 (52%)	42.60 ± 12.52	0.001 Bonferroni retired v not employed *p* = .001
Employed/self employed	38 (28%)	38.26 ± 12.37	
Not in paid employment	27 (20%)	31.69 ± 12.33	
Gave up work due to illness			
Yes	53 (39%)	32.34 ± 10.76	< 0.0001
No	82 (61%)	43.63 ± 12.51	
Education (missing *N* = 2)			
Secondary	51 (38%)	40.91 ± 13.71	0.236
Further	17 (13%)	35.59 ± 10.48	
University	32 (24%)	35.78 ± 12.55	
Professional qualifications	33 (25%)	41.52 ± 13.46	
Caregiving responsibilities			
Yes	88 (65%)	37.74 ± 12.79	0.076
No	47 (35%)	41.92 ± 13.21	
Caregiving responsibilities (for children/grandchildren only)			0.413
Yes	40 (30%)	37.78 ± 14.03	
No	95 (70%)	39.79 ± 12.63	

^a^Ethnicity is not included as 96% of the population identified as White British

^b^Total scores on final modified scale, post item reduction

### Acceptability of PRRS

Missing data rate for the PRRS was extremely low, 0.6% at baseline, 0.5% at Time 2 and 0.6% at Time 3. Missing data were distributed across 10 of the 28 Likert-scale items at baseline, 7 of the 28 items at Time 2 and 10 at Time 3 with no single item having more than 4 missing responses. The highest rate of missing data for a single item at any time point was 7% (an employment question with 26/28 possible responses); for core questions with the larger sample, the highest rate of missing data for a single item at any time point was 3% (threshold for investigation was > 15% missing data).

At baseline, 60 participants completed questionnaires online, 75 completed on paper but only 61 recorded the time taken to complete the PRRS. Time to complete online ranged from 1.87 to 14.05 min with 1 extreme outlier at 34.78 min (mean excluding outlier = 4.71 min, SD = 2.21 min). On paper, time to complete ranged from 2 to 20 min with one extreme outlier at 30 min (mean excluding outlier = 8.52 min, SD = 4.22 min).

Remaining analyses and results are presented separately for the PRRS core items and the jobs and career subscale, which is only completed by participants currently in paid employment.

### PRRS core items

#### Precision and item reduction

Five items had an unacceptably low level of correlation with other items and low (< 0.3) corrected item-total correlations in reliability analysis. In addition, one of these did not meet the measure of sampling adequacy (set at 0.7). These five underperforming items were removed from further analysis. Four of the retained items had z-scores of skewness >ǀ4ǀ; however, their performance in terms of relationship to other items corrected item-total correlation and/or conceptual significance to the measure ensured their retention at this stage. No item correlated *r* > 0.8 with any other item.

None of the remaining individual items had missing data > 15% or floor effects exceeding 70% (range 1.5–41.5%, on 12/16 items less than 20% of responses were the minimum ‘0’ response option). One item showed above threshold ceiling effects (74% ‘my family have to help me financially’, note this item is reverse scored; a response of  ‘not at all’ receives a score of ‘4’). Scores of maximum ‘4’ ranged from 7.4 to 74%; 4/16 items had less than 20% of responses at maximum ‘4’.

Total scores on the modified PRRS Core-16 (*N* = 135) ranged from 10 to 64 (possible range 0–64), with mean, 39.20, SD 13.04, with skewness = − 0.08 (SE = 0.204). No participants had minimum scores. 2 participants (1.5%) had maximum scores (threshold for ceiling effects set at 15%).

### Exploratory factor analysis

**Fig. 2:**
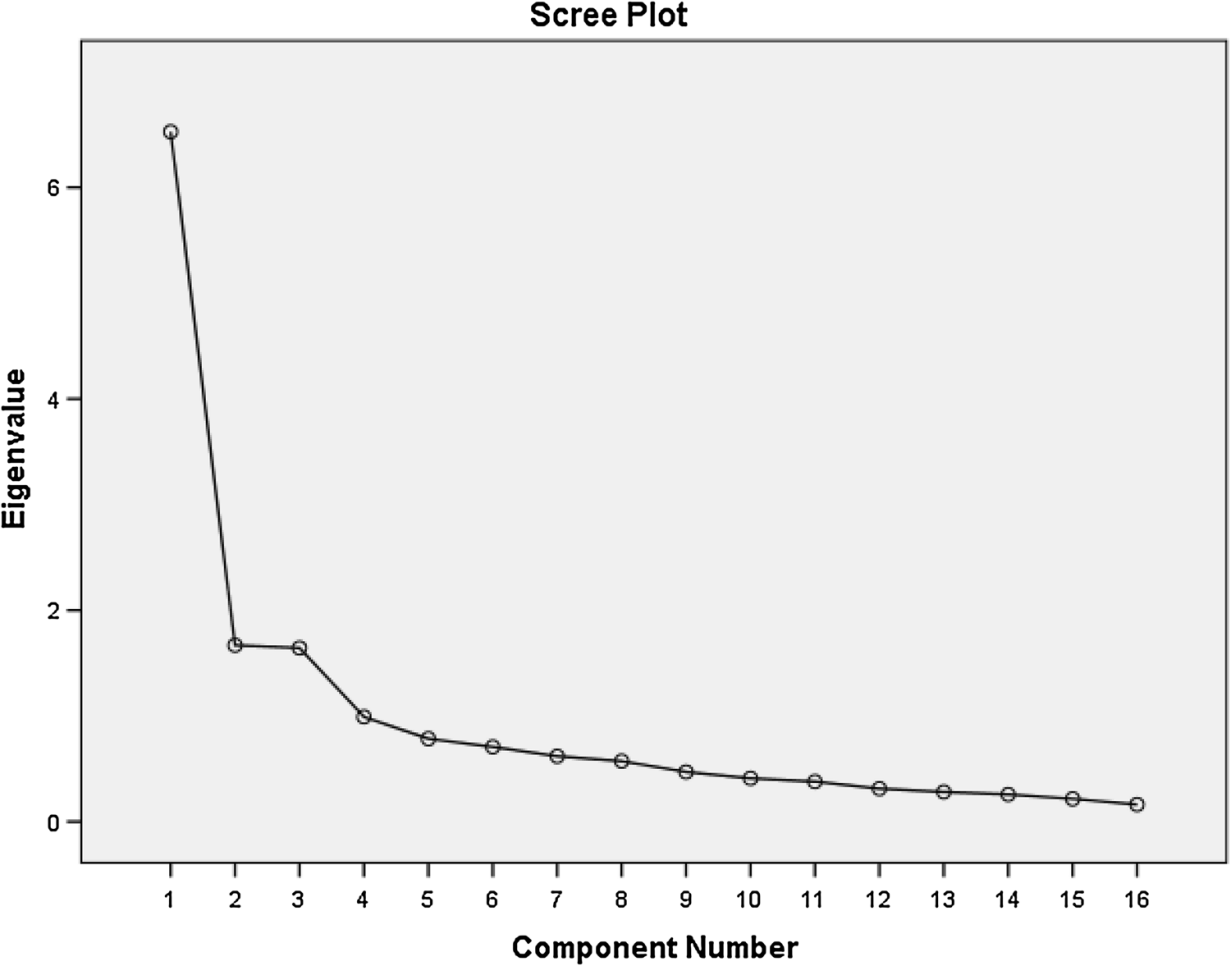
Scree plot

Following listwise deletion based on all variables, *N* = 130. Bartlett’s test of sphericity was significant at < 0.0001 and the KMO value was 0.850 confirming that the data were suitable for factor analysis. MSA for all variables was > 0.7 suggesting adequate communality with other variables. 3 Eigenvalues were greater than 1 explaining 40.8, 10.4 and 10.3% of variance respectively (total variance explained 61.5%). The scree plot (Fig. 2) confirmed that 3 factors should be retained corresponding to family well-being (Factor 1), financial well-being (Factor 2) and responsibilities and social life (Factor 3). All items except one loaded clearly on one factor. One item (PL9: “The way I see myself within the family has changed because of my illness”) cross loaded on Factor 1 (0.433) and Factor 3 (− 0.428). This item was retained for the family well-being subscale. The items and their loadings are shown in Table 2.

**Table 2:** Item factor loading

	Item	Factor 1	Factor 2	Factor 3
PL1	My illness interferes with performing my responsibilities at home (e.g. cooking, cleaning, gardening, DIY)			− 0.790
PL3	I am less able to fulfil my caregiving responsibilities (e.g. looking after children, grandchildren, another adult, pets)			− 0.892
PL4	I have less patience for my caregiving responsibilities (e.g. looking after children, grandchildren, another adult, pets)			− 0.674
PL5	I feel sad that my illness forces me to miss out on doing things with my children and/or other family members			− 0.681
PL6	I worry about the impact of my illness on my partner (or the person who is my main support)	0.803		
PL7	I worry about the impact of my illness on my children and/or other family members	0.801		
PL8	I worry about the impact of my illness on people that I normally provide support to (e.g. friends, neighbours, parents and/or grandchildren)	0.774		
PL9	The way I see myself within the family has changed because of my illness	0.433		− 0.428
PL11	I worry how my family will cope in the future	0.872		
PL13	I socialise less because of my illness			− 0.653
FT3	I worry about the financial problems I will have in the future as a result of my illness or treatment		0.561	
FT11	I feel in control of my financial situation		0.500	
PF3	My family gives up things because of the financial impact of my illness		0.618	
PF4	My family have to help me financially		0.798	
PF5	The additional costs of my illness are more than I thought they would be (e.g. travel and parking, heating, healthy eating, supplements, non-prescription medication, paying for help at home)		0.686	
PF6	I have difficulty meeting the additional costs of my illness		0.874	

Reliability analyses were conducted for the three potential subscales using only the final items within the subscales. Cronbach’s α for family well-being was 0.87 (*N* = 132, corrected item-total correlation range 0.60–0.76); for responsibilities and social life, 0.84 (*N* = 133, CITC range 0.50–0.71) and for financial well-being 0.79 (*N* = 135, CITC range 0.47–0.71). Inter-item and inter-subscale correlations are shown in Table 3.

**Table 3:** Inter-item correlations and correlation between subscale scores

Subscale	Mean (SD) Range	Inter-item correlation range	Correlations between subscale scores		
			Family well-being	Responsibilities and social life	Financial well-being
Family well-being (5 items, possible score 0–20)	8.38 (5.57) 0–20	0.479–0.670 *N* = 132	1.00		
Responsibilities and social life (5 items, possible score 0–20)	12.85 (5.12) 0–20	0.324–0.665 *N* = 133	0.563 *N* = 135	1.00	
Financial well-being (6 items, possible score 0–24)	17.97 (5.01) 5–24	0.211–0.610 *N* = 135	0.544 *N* = 135	0.493 *N* = 135	1.00

### Internal consistency

Cronbach’s α for the modified PRRS total score at baseline (*N* = 131, listwise deletion based on all variables) was 0.90 (CITC range 0.43–0.72); at T2 (*N* = 124) α = 0.90 (CITC range 0.37–0.73); and at T3 (*N* = 112) α = 0.91 (CITC range 0.43–0.76). In no case was α increased by the deletion of an item.

#### Criterion and convergent validity

As predicted, total scores on the PRRS correlated strongly and negatively with scores on the SDI (*N* = 135, *r* = − 0.77). We did not find the floor effects reported elsewhere for SD-16 scores [27]; however, the distribution was skewed in that direction. As a precautionary measure, we categorised participants as distressed or not distressed based on a cut-off threshold of ≥ 10/44 as recommended by the instrument authors [21]. 58 (43%) participants were categorised as distressed. We then used this dichotomous variable in a point-biserial correlation with total PRRS score. As expected, the correlation was strong; *N* = 135, *r* = − 0.65. Correlations between the PRRS subscales and most appropriately matched subscales of the SDI were also moderate to strong: PRRS responsibilities and social life with SDI everyday living *r* = − 0.71; PRRS financial well-being with SDI money matters *r* = − 0.65; PRRS family well-being with SDI self and others *r* = − 0.52. PRRS scores showed the predicted correlations with FACT-G (*N* = 135, *r* = 0.65) and WHOQOL-BREF (*N* = 135, *r* = 0.65).

### Test–retest reliability

The median number of days between baseline and T2 was 8 (mode 7, range 3–21). Total PRRS scores showed good test–retest reliability (two-way random, absolute agreement) (*N* = 128, ICC = 0.86, 95% CI 0.80–0.90) as did the three subscales responsibilities and social life (*N* = 128, ICC = 0.83, 95% CI 0.77–0.88), family well-being (*N* = 128, ICC = 0.77, 95% CI 0.68–0.83) and financial well-being (*N* = 128, ICC = 0.86, 95% CI 0.81–0.90). Weighted kappa scores for individual items were all in the acceptable range (*κ* ≥ 0.4) and ranged from 0.44 to 0.63.

### Sensitivity to change

The median number of days between baseline and T3 was 66 (mode 60, range 56–117). Table 4 shows change over time in the anchor variable GF7 (I am content with the quality of my life right now). 47.5% of participants reported no change, 26.25% of participants reported an improvement by one or more points and the same proportion reported decline by one or more points. Participants whose GF7 score improved showed significant improvement in their PRRS scores (*p* = .008), those reporting decline on GF7 showed significant decline on PRRS (*p* = .001). As predicted, PRRS scores did not change significantly for those participants reporting no change on GF7 (*p* = .891). The groups do not differ at baseline. PRRS change scores correlated significantly with change on the anchor variable (*r* = 0.42) and with change on other measures: FACTG (*r* = 0.48) SDI (*r* = − 0.42) and WHOQOL-BREF (*r* = 0.32).

**Table 4:** PRRS change by change on anchor variable GF7 (I am content with the quality of my life right now)

I am content with the quality of my life right now	*N*	Baseline PRRS mean (SD)	Mean PRRS change (SD)	*p*	Effect size^a^
Improved by one point or more	31	39.21 (13.87)	− 3.88 (7.58)	0.008	− 0.28
Unchanged	56	39.52 (13.67)	− 0.12 (6.72)	0.891	− 0.01
Worsened by one point or more	31	40.58 (12.10)	4.55 (6.79)	0.001	0.38

^a^Mean change score/SD mean baseline score

### PRRS jobs and career subscale

The jobs and career subscale was completed by just 33, 30 and 28 patients (31, 29, 25 provided complete data) at the different time points, respectively; too few for reliability analyses. Table 5 shows baseline summary statistics for the items that make up the scale. The item PE3 had a particularly weak pattern of correlation with other subscale items (*N* = 33, *r* = 0.03–0.32) and two items (PE3 and PE7) demonstrated ceiling effects; however due to the small number of participants completing the scale, we will not eliminate items at this stage.

**Table 5:** Baseline descriptive statistics for items on the jobs and career subscale (*N* = 33)

	Item	Missing data (%)	Mean ± SD	Median/mode	|z-score skew|	% floor (score 0)	% ceiling (score 4)
PE2	I have reduced my working hours because of my illness^a^	0	2.06 ± 1.66	3.0/0	0.57	33.3	24.2
PE3	My working hours are flexible to accommodate my treatment and appointments	0	3.00 ± 1.44	4.0/4	2.97	12.1	57.6
PE4	I feel I am able to do my job as well as I would like	0	2.30 ± 1.45	3.0/3	1.39	21.2	21.2
PE5	I worry that my illness will impact my employment in the future (including return to work)^a^	0	1.64 ± 1.34	2.0/2	0.76	27.3	12.1
FT9	I am concerned about keeping my job and income^a^	0	1.79 ± 1.52	2.0/0	0.38	30.3	18.2
PE6	I feel that my illness has limited my career^a^ opportunities	0	2.48 ± 1.77	3.0/4	1.43	30.3	48.5
PE7	I feel supported by my employer	6.06	3.39 ± 1.12	4.0/4	4.21	3.2	71.0

^a^Items are reverse scored

## Discussion

We report the development and validation of the PRRS, a patient self-report measure of the impacts of cancer and treatment on family, financial and occupational roles and responsibilities. The format and structure are compatible with the FACIT measurement system. The scale was developed with advanced cancer patients and found to have very good reliability and validity in field testing. Although developed and validated on people with advanced cancer, the scale was developed with the intention to be sufficiently generic and appropriate for use in all stages of cancer. Indeed, due to the nature of the topics covered in the PRRS (i.e. family, finances, employment), there is potential for use in other chronic conditions. The scale is currently undergoing field testing with patients with early stage cancer. Extensive qualitative work to establish content validity would be required before any field testing in other chronic conditions would be possible. This should be a topic for further research.

The PRRS appears to be acceptable to participants both in terms of time to complete and the very low missing data rate in total and per item. Missing data on the final scale appear to be at random and are therefore assumed not to be indicative of a problem with any particular item. No participants recorded minimum scores on the final scale and only 2/135 recorded maximum scores.

Factor analysis of the Core-16 items revealed a three-factor solution corresponding to three potential subscales: family well-being, financial well-being, and responsibilities and social life. All demonstrated good internal consistency as does the PRRS-16 total score. Future studies will seek to confirm the factor structure and factorial invariance. The PRRS-16 total score showed the predicted strength of correlations with measures for criterion and convergent validity and good test–retest reliability. The scale demonstrated sensitivity to change; those patients showing improvement on the anchor variable showed significant improvement in PRRS scores, and those declining on the anchor variable showed significant decline in PRRS scores. Though significant, the effect size in both groups was small to medium. The magnitude of the effect is likely related to our choice of anchor variable and that we set just one point shift as the threshold for change on the anchor. As reported elsewhere [28] the absolute PRRS change was greater in those reporting worsening on the anchor variable than those reporting improvement.

We believe that there is utility in a total score for the PRRS, particularly when the scale is being used as part of a clinical discussion for straightforward monitoring of global change over time and as a way to quickly flag patients who might be experiencing difficulties and for whom further discussion and investigation might be appropriate. That said, the subscale scores may provide useful indication of how different domains are impacted for different individuals, allowing for more tailored approach to supportive interventions. Interpreting the profile of subscale scores rather than a total score is likely to be more appropriate and useful in some circumstances [29, 30].

We did not make a priori hypotheses on group differences on either the PRRS total score or scores on separate domains. We found that age was a significant factor with significant post hoc contrasts between those over 65 years and those 50 years or younger. Those patients already retired had significantly higher scores than those not in paid employment (but not those currently working) and those who had been forced to give up work due to illness had significantly lower scores than those who had not (either because they were not working or had continued to work). Scores for those who had caregiving responsibilities were lower than for those who did not, but not significantly so. A more nuanced exploration of the role of age and responsibilities to self and others is beyond the remit of this paper but will be presented elsewhere.

We indicated in the introduction that excellent, well-validated measures such as the SDI [7, 8] and COST [5, 6] exist. We believe that the PRRS provides a useful scale with more comprehensive coverage. For example, the SDI is not intended to provide a full assessment of jobs and careers; likewise the COST was not developed to measure family function and responsibilities. The PRRS has been developed following FACIT convention with the intention that it will sit within the FACIT measurement system and can be used alongside the FACT-G and or/other FACIT measures to provide comprehensive assessment.

### Limitations

The main limitation relates to sample size for the validation study, particularly the jobs and careers subscale. While we have sufficient participant numbers for the factor and reliability analysis of the core questionnaire items, we were unable to evaluate the jobs and careers subscale fully. A further validation study is currently underway to address this shortfall.

We chose to develop the measure in patients with stage III/IV cancer; the stimulus for the programme of research being the increase in numbers of people living with cancer as a chronic condition. We also developed the measures in a limited number of tumour groups which although chosen to ensure a spread of key patient characteristics such as age, inevitably resulted in some sampling bias. For example two of the four cancer sites (breast and gynaecology) recruited only females resulting in a disproportionate number of female participants (77%). We did not have access to performance status scores for patients; this would be a useful group comparison in future research.

In any development study, choices are made regarding participant characteristics which limit the immediate generalizability of the findings. However, the PROACT measures were developed with an intention to be sufficiently generic for application to all disease stages and tumour types. Further validation will ensure that the measures are sufficiently generic for use in clinical trials and realworld studies.

### Conclusion

The PRRS was developed to capture broad ‘real life’ impacts of cancer and its treatment and has potential use for both research and practice. For example, it may be used in the assessment of quality of life during the evaluation of new treatments in clinical trials and real world studies. It is potentially useful therefore to regulatory authorities scrutinising data from clinical trials and to policy makers when determining ‘costs’ of different treatments. The scale could also aid clinical conversations around treatment and intervention, with a growing body of evidence demonstrating the value of using PROMs in this way [31–35]. This initial evaluation shows that the PRRS is psychometrically robust with potential to inform the evaluation of novel therapies and to help drive the development of ameliorative interventions for the enhancement of extended survivorship.

## Electronic supplementary material

Below is the link to the electronic supplementary material.

Supplementary material 1 (DOCX 14 KB)
