# Supplementary material for: Development and validation of the patient roles and responsibilities scale in cancer patients
Source: Qual Life Res. 2018 Jul 26;27(11):2923–34. doi: 10.1007/s11136-018-1940-2 (PMC6208586; doi:10.1007/s11136-018-1940-2)
Supplement: Supplementary file 1 — Supplementary material 1 (DOCX 14 KB) [file 11136_2018_1940_MOESM1_ESM.docx]

Supplementary File S1: Participant demographics Studies 1 and 2

| Study 1 | | | | | |
| --- | --- | --- | --- | --- | --- |
|  | Overall (N=24) | Gynae (N=9) | Melanoma (N=9) | Lung (N=6) |  |
| Age Range (median) | 39-84  (62yrs) | 44-84  (64yrs) | 37-69  (59yrs) | 53-79  (63.5yrs) |  |
| Male/Female | 8 (33%)/16 (67%) | - | 6M 3F | 2M 4F |  |
| Time since diagnosis | <1yr = 11  1-2yrs = 5  >2yrs = 8 | <1yr = 5  1-2yrs = 1  >2yrs = 3 | <1yr = 3  1-2yrs = 2  >2yrs = 4 | <1yr = 3  1-2yrs = 2  >2yrs = 1 |  |
| Study 2 | | | | | |
|  | Overall (N=20) | Gynae (N=6) | Melanoma (N=7) | Lung (N=4) | Breast (N=3) |
| Age Range (median) | 39-79  (63.5yrs) | 44-73  (61.5yrs) | 39-79  (69yrs) | 45-76  (59.5yrs) | 39-50  (39yrs) |
| Male/Female | 5 (25%)/15 (75%) |  | 3M 4F | 2M 2F | 0M 3F |
| Time since diagnosis | <1yr = 8  1-2yrs = 1  >2yrs = 11 | <1yr = 3  1-2yrs = 1  >2yrs = 2 | <1yr = 1  1-2yrs = 0  >2yrs = 6 | <1yr = 2  1-2yrs = 0  >2yrs = 2 | <1yr = 2  1-2yrs = 0  >2yrs = 1 |
